# Serum Albumin as an Independent Predictor of Long-Term Survival in Patients with Recurrent and Metastatic Head and Neck Squamous Cell Carcinoma Treated with Nivolumab

**DOI:** 10.3390/jcm13092456

**Published:** 2024-04-23

**Authors:** Shinsuke Suzuki, Yukie Taguchi, Takuro Kitabayashi, Nobuko Sato, Haruka Kaya, Tomoe Abe, Tentaro Endo, Hitomi Suzuki, Yohei Kawasaki, Takechiyo Yamada

**Affiliations:** Department of Otorhinolaryngology & Head and Neck Surgery, Akita University Graduate School of Medicine, Akita 010-8543, Japan

**Keywords:** immunotherapy, neoplasm metastasis, neoplasm recurrence, local, nivolumab, prognosis, serum albumin, squamous cell carcinoma of head and neck, survival analysis

## Abstract

**Background**: Nivolumab has been shown to improve the overall survival (OS) of patients with recurrent and metastatic head and neck squamous cell carcinoma (R/M HNSCC). However, there is a need to identify factors associated with long-term survival (beyond 2 years) in these patients. This study investigated the relationship between pretreatment factors and long-term survival in patients with R/M HNSCC treated with nivolumab. **Methods**: Forty-nine patients with R/M HNSCC who were treated with nivolumab were retrospectively reviewed. Baseline characteristics, clinical data, and survival outcomes were evaluated. Univariate and multivariate analyses were performed to identify factors associated with long-term survival (OS ≥ 2 years). **Results**: The median OS in the overall cohort was 11.0 months, and the 2-year survival rate was 34.7%. Long-term survivors (OS ≥ 2 years) had significantly higher proportions of patients with Eastern Cooperative Oncology Group (ECOG) performance status (PS) scores of 0 or 1, serum albumin levels ≥ 3.5 g/dL, and neutrophil-to-eosinophil ratio (NER) < 32.0 compared to non-long-term survivors. On multivariate analysis, serum albumin levels ≥ 3.5 g/dL, in addition to ECOG-PS score of 0 or 1, were independent predictors of long-term survival. **Conclusions**: Pretreatment serum albumin levels may be useful for predicting long-term survival in R/M HNSCC patients treated with nivolumab.

## 1. Introduction

Improving the prognosis of patients with head and neck squamous cell carcinoma (HNSCC) through technological advances in surgery and radiation therapy as well as through the development of platinum-based chemotherapy is a contemporary research hotspot [1,2]. However, over half of these patients experience local recurrence or distant metastasis [3,4]. Recurrent and metastatic HNSCC (R/M HNSCC) is often challenging to treat, and as a result the prognosis of these patients is extremely poor [5]. Therefore, development of therapies for local recurrence and distant metastasis is imperative to prolong the survival of patients with HNSCC.

Immune checkpoint inhibitors (ICIs) inhibit cancer growth and metastasis by blocking the immune escape of cancer cells [6]. ICIs have revolutionized the treatment of head and neck cancer and currently play a central role against R/M HNSCC [7]. In a phase III trial for platinum-refractory R/M HNSCC (CheckMate-141), nivolumab extended overall survival (OS) compared to conventional chemotherapy [8]. Thus, nivolumab is now considered a first-line therapy for platinum-resistant R/M HNSCC. Platinum remains the primary drug for treating HNSCC. Therefore, nivolumab, which is the first-line treatment for platinum-resistant R/M HNSCC, plays a remarkable role in the treatment of head and neck cancer [9,10].

Tail plateau effect is known to occur during ICI treatment, regardless of the type of cancer [11,12]. The tail plateau effect refers to the phenomenon wherein the OS and progression-free survival (PFS) curves nearly stop falling after a certain point, conferring a long-term PFS benefit that was believed to be impossible with conventional therapy [13]. Moreover, the tail plateau effect has also been observed in R/M HNSCC where the number of mortalities decreased after 2 years in patients treated with nivolumab, and many patients survived longer thereafter [14,15,16]. Hence, in R/M HNSCC patients treated with nivolumab, survival for 2 years is significant.

The long-term follow-up results of CheckMate-141 revealed a 2-year survival rate of 16.9%, as reported by Ferris in 2018 [14]. This result is noteworthy when compared to conventional chemotherapy. However, it should be noted that many patients do not survive beyond 2 years, indicating the need for further improving the OS of R/M HNSCC patients treated with nivolumab. Thus, a more efficient use of nivolumab is crucial for this purpose.

Patient selection using biomarkers is one of the most important tools for efficient drug use. Previous studies have investigated the prognostic relevance of various patient characteristics and biomarkers in patients undergoing ICI treatment including nivolumab [17,18]. These factors can be broadly divided into two categories: (1) post-treatment factors, which can only be determined after drug administration, such as response rate and the presence of immune-related adverse events (irAEs) [19,20]; and (2) pretreatment factors, which can be determined before drug administration, such as patient background factors such as Eastern Cooperative Oncology Group (ECOG) Performance Status (PS) [21] and PD-L1 expression in cancer tissue. The latter is particularly important for nivolumab, a PD-1 inhibitor [22].

The use of pretreatment factors is particularly important for efficient drug treatment as it can help prevent wastage of time and money on ineffective and wasteful therapies. Furthermore, it can help minimize irAEs [23], which occur early in dosing and are often severe. Therefore, several studies have sought to identify pretreatment factors that predict prognosis in R/M HNSCC patients treated with nivolumab [24,25,26]. However, no factors have been found to directly contribute to long-term survival beyond 2 years in R/M HNSCC patients treated with nivolumab.

Serum albumin level has been identified as a prognostic factor in many malignant diseases [27,28,29]. Specifically, pretreatment serum albumin level was recently found to be useful for predicting the response to ICI treatment in various carcinomas, including head and neck cancer [30,31,32]. However, whether pretreatment serum albumin levels are associated with long-term survival in patients with R/M HNSCC treated with nivolumab has not yet been examined. Therefore, this study aimed to examine whether various patient pretreatment factors, including serum albumin levels, are associated with long-term survival beyond 2 years in patients with R/M HNSCC treated with nivolumab.

## 2. Materials and Methods

### 2.1. Patients and Data Collection

We retrospectively reviewed the medical records of consecutive patients with R/M HNSCC who had received nivolumab treatment at the Department of Otorhinolaryngology–Head and Neck Surgery of the Akita University Hospital (Akita, Japan) between 1 October 2017, and 31 August 2021. The cutoff date was 1 September 2023, and only patients who had been on nivolumab for at least 2 years were eligible for inclusion. This study was approved by the Institutional Review Board of Akita University Hospital (No.: 2873) and conducted according to the principles of the Declaration of Helsinki. Given the retrospective nature of the study, the requirement for informed consent of patients was waived; however, patients were allowed to decline the use of their clinical records for research (opt-out consent provision). The study began on 1 September 2022 and participant data were collected from the start of the study to 7 December 2022. All patient data are coded to ensure the privacy of the subjects and data confidentiality.

Patients with OS of ≥2 years were defined as long-term survivors because in the phase III trial CheckMate 141 [14] and a study based on real-world data [33], mortality of patients treated with nivolumab decreased after 2 years, and the 3- and 5-year survival rates were approximately the same during the long-term follow-up.

The following patient characteristics and clinical data were collected from our institutional medical records immediately before the start of nivolumab treatment: age, ECOG PS, sex, body mass index (BMI), primary tumor site, treatment target site, presence of diabetes mellitus, previous radiotherapy to the primary tumor, platinum-refractory carcinoma, previous exposure to cetuximab, and laboratory data, i.e., serum albumin (Alb), neutrophil-to-lymphocyte ratio (NLR), and neutrophil-to-eosinophil ratio (NER).

The clinical data were classified as low/high or normal using the following cutoff points based on previous studies: BMI 18.5 [34], serum albumin level 3.5 g/dL [35,36], NLR 5.0 [37,38], and NER 32.0 [24].

Platinum-refractory carcinoma was defined as carcinoma that recurred or progressed within 6 months of final administration of platinum-based drugs. The best overall response (BOR) of patients was classified as follows: complete response (CR), partial response (PR), stable disease (SD), and progressive disease (PD). Progression and response were evaluated based on the Response Evaluation Criteria in Solid Tumors (RECIST, version 1.1). irAEs were assessed according to the Common Terminology Criteria for Adverse Events (version 4.0).

### 2.2. Statistical Analysis

Dichotomous variables, such as the baseline characteristics, were compared using Fisher’s exact test. Continuous variables were represented as median (range) and between-group differences were assessed using the Wilcoxon rank-sum test. OS curves were constructed using the Kaplan–Meier method, and between-group differences were evaluated using the log-rank test. Univariate logistic regression analysis was conducted for each candidate variable to calculate the odds ratios for association with long-term survival. Variables that were associated with a *p*-value < 0.05 in the univariate analysis were included in the multivariable logistic regression analysis to identify independent factors related to long-term survival with nivolumab. Multivariate logistic regression analysis was evaluated using Hosmer and Lemeshow test; its goodness of fit was found to be adequate (*p* = 0.316). Statistical analyses were performed using EZR, version 4.0.0 (Saitama Medical Center, Jichi Medical University, Saitama, Japan). Moreover, *p*-values < 0.05 were considered indicative of statistical significance.

## 3. Results

### 3.1. Baseline Characteristics

The study included 49 patients with R/M HNSCC who were treated with nivolumab. The median follow-up in this cohort was 12.4 months (1.5–59.2). Of the 49 patients, 17 were long-term survivors (OS ≥ 2 years) and 32 were non-long-term survivors (OS < 2 years). Five (10.2%) of the long-term survivors continued nivolumab treatment until the cutoff date. Table 1 summarizes the baseline demographic and clinical characteristics of all patients. There were significant differences between long-term survivors and non-long-term survivors in terms of ECOG-PS (*p* = 0.020), serum albumin (*p* = 0.006), and NER (*p* = 0.007).

### 3.2. Survival Outcomes

The median OS for all 49 patients was 11.0 months (95% CI 6.3–19.4) and the 2-year survival rate was 34.7% (95% CI 21.8–47.9). Median OS for long-term survivors was not reached and the 2-year survival rate was 94.1% (95% CI 65.0–99.1). Non-long-term survivors had a median OS of 6.2 months (95% CI 3.1–9.4) and the 2-year survival rate was 0.0% (95% CI not available [NA]-NA).

**Figure 1:**
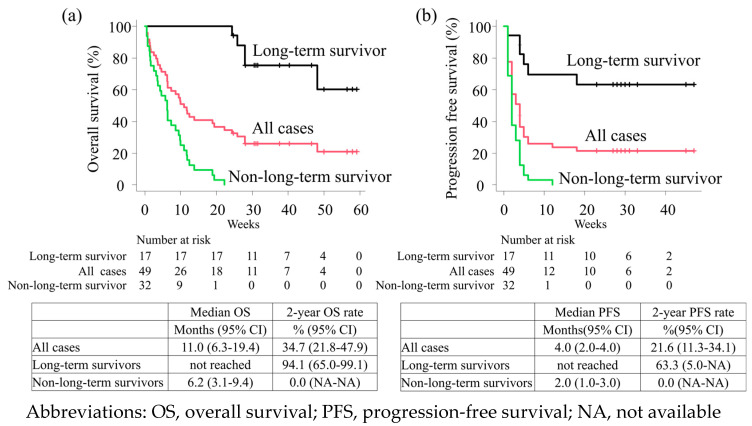
Kaplan–Meier survival curves of OS (**a**) and PFS (**b**) for all patients, long-term-survivors, and non-long-term survivors.

The overall median PFS was 4.0 months (95% CI 2.0–4.0) and the 2-year PFS rate was 21.6% (95% CI 11.3–34.1). The median PFS among long-term survivors was not reached (95% CI 5.0–NA) and the 2-year PFS rate was 63.3% (95% CI 35.8–81.6). The median PFS for non-long-term survivors was 2.0 months (95% CI 1.0–3.0) and the 2-year PFS rate was 0.0% (95% CI NA–NA). OS and PFS for all patients and subgroups are displayed in Figure 1.

### 3.3. Treatment Response

Outcomes of nivolumab therapy are summarized in Table 2. The overall objective response rate (ORR) was 58.6% and the disease control rate (DCR) was 44.9% (CR 14.3%, PR 20.4%, SD 10.2%, and PD 55.1%). The ORR in long-term survivors was 88.2% and the DCR was 100% (CR 41.2%, PR 47.1%, SD 11.8%, and PD 0%). The ORR in non-long-term survivors was 6.3% and the DCR was 15.6% (CR 0%, PR 6.3%, SD 9.4%, and PD 84.4%). ORR and DCR were significantly higher in long-term survivors than in non-long-term survivors (*p* < 0.001 for both).

### 3.4. Immune-Related Adverse Events

Overall, irAEs occurred in 12 of 49 patients (28.9%). Hypothyroidism was the most common irAE (*n* = 8), followed by hypoparathyroidism (*n* = 4) and dermatitis (*n* = 1). There was one case of enterocolitis, but it was Grade 3. Seven of 17 (41.2%) long-term survivors and 5 of 32 (15.6%) non-long-term survivors developed irAEs, and there was no significant between-group difference in this respect (*p* = 0.0769). The irAEs are summarized in Table 3.

### 3.5. Univariate and Multivariate Analysis of Factors Associated with Long-Term Survival

The results of univariate and multivariate analyses are shown in Table 4. On univariate logistic regression analysis, ECOG-PS 0 or 1 (OR = 12.4, 95% CI: 1.47–106.0, *p* = 0.0208), NER of <32 (OR = 6.2, 95% CI: 1.63–23.6, *p* = 0.0.00746), and serum Alb of ≥3.5 mg/dL (OR = 8.5, 95% CI: 1.66–43.4, *p* = 0.0101) were significantly associated with long-term survival.

In multivariate analysis, only ECOG-PS scores of 0 or 1 (OR = 11.9, 95% CI: 1.21–116.0, *p* = 0.0339) and serum Alb of ≥3.5 g/dL (OR = 6.09, 95% CI: 11.02–36.40, *p* = 0.0475) were significantly associated with long-term survival. In contrast, NER of <32 (OR = 4.27, 95% CI: 0.934–19.50, *p* = 0.0613) showed no significant association with long-term survival.

## 4. Discussion

To the best our knowledge, this is the first study to demonstrate pretreatment serum albumin levels as an independent predictor of long-term OS (OS ≥ 2 years) in R/M HNSCC patients treated with nivolumab.

Serum albumin is a convenient marker of visceral protein function. Moreover, albumin synthesis is suppressed by malnutrition and inflammation. Therefore, serum albumin is commonly used to assess nutritional status, disease severity, disease progression, and prognosis [39]. A plethora of studies have examined the prognostic value of serum albumin levels. In hospitalized patients, low serum albumin level was identified as a risk factor for nutrition-related complications [40]. Furthermore, low serum albumin concentration has been shown to adversely affect patient prognosis and increase the risk of death [41,42]. Notably, low pretreatment serum albumin levels in cancer patients are associated with poor survival [43,44,45], and low serum albumin levels are associated with poor response to various chemotherapies and tyrosine kinase inhibitors (TKIs) [46,47]. More recently, serum albumin levels were demonstrated to predict the prognosis of ICI therapy [30,32].

Albumin is the most abundant transport protein in blood and it transports a myriad of anti-tumor proteins to the tumor site [48]. Moreover, albumin is considered to be one of the covariates of IgG-based antibody drug clearance, and low serum albumin has been shown to correlate with increased clearance of monoclonal antibodies in various diseases, including malignancies [49,50,51]. Turner et al. discovered that patients with clinical features of cancer cachexia, such as excessive weight loss and albumin loss, had a faster clearance of pembrolizumab, and faster clearance was associated with shorter OS in patients with advanced melanoma and non-small cell lung carcinoma [52].

Nivolumab is a human IgG4 monoclonal antibody like pembrolizumab. Therefore, in the presence of low serum albumin, nivolumab may not be adequately transported in the blood and may have an even faster clearance from the body. Therefore, nivolumab is not completely effective. These reasons may explain why low serum albumin led to worse OS in this study. In this study, we also examined the prognostic relevance of NLR and NER, which can be calculated from peripheral blood indices. NLR and NER, like albumin, are believed to reflect host nutrition and immunity and have been reported as predictors of treatment response in ICI [53,54].

However, in the present study, these factors were not found to be independent predictors of long-term survival in R/M HNSCC patients treated with nivolumab, which may be attributable to the small sample size. Indeed, NER was associated with long-term survival in univariate analysis. A future study with a larger sample size is required to obtain further evidence.

Considering the mechanism of action of ICIs, information obtained from tumor tissue cannot be overlooked as a predictor of efficacy. In addition to PD-L1 expression in tumor tissue, other factors in the tumor microenvironment such as tumor mutation burden, tumor-infiltrating lymphocytes, and hypoxia have been reported as predictors of therapeutic response to ICIs, including nivolumab [22,55,56]. Since these factors measured from tumor tissue directly reflect tumor immunity, they are expected to be more robust predictors of the therapeutic efficacy of ICIs. However, measurement of these biomarkers requires expensive equipment. In addition, tissue collection requires invasive procedures or may not be possible in cases of distant metastases, which is a major limitation in clinical practice.

Additionally, in the multivariate analysis, pretreatment PS also demonstrated an independent association with long-term survival. PS is a commonly used and convenient prognostic factor for ICI therapy [26,57]. However, assessment of PS is subject to interobserver variability [58]. Conversely, serum albumin level is an objective and more reliable marker.

RECIST score and irAEs are strong prognostic factors for ICI therapy that can be considered after the start of treatment [26,59]. Herein, BOR was also significantly better in the long-term survival group than in the non-long-term group, suggesting that BOR is useful in determining the continuation of treatment. However, there were no significant differences regarding irAEs between long-term and non-long-term survivors in this study. Nonetheless, there are known differences in subjective Aes such as fatigue between patients and medical providers [60,61,62] and it is possible that subjective AEs were not adequately captured in this study. We believe that a more careful observation of AEs is needed in future studies.

However, as noted above, the RECIST score and presence of irAEs are measurable only after the initiation of treatment. Although they are strong predictors of ICI efficacy, they cannot be assessed until the drug is used. Therefore, these factors are only useful for decision-making regarding the continuation of ICI treatment.

Considered together, serum albumin levels, which can be easily and objectively assessed before treatment, can be a useful marker for predicting long-term survival in patients with R/M HNSCC treated with nivolumab.

Some limitations of this study should be considered while interpreting the results. First, this was a single-center, retrospective study with a relatively small sample size and a short follow-up period. Second, tumor-related factors, such as PD-L1 expression in tumor tissue, were only measured in a subset of patients. Therefore, although PD-L1 expression in tumors is an important predictor of the efficacy of nivolumab therapy, we were unable to examine its impact on clinical outcomes. Furthermore, the relationship between PD-L1 expression and serum albumin could not be assessed. Therefore, to optimize drug therapy for R/M HNSCC, the present findings need to be validated in a large prospective study with long-term follow-up. In addition, further studies on biomarkers in nivolumab-treated patients are needed.

## 5. Conclusions

In this cohort of patients with R/M HNSCC treated with nivolumab, pretreatment serum albumin levels ≥3.5 g/dL were identified as an independent predictor of long-term OS (OS ≥ 2 years), along with an ECOG performance status score of 0 or 1. These findings suggest that pretreatment serum albumin levels may serve as a valuable prognostic marker for determining the long-term benefit of nivolumab therapy. Further studies are required to confirm this result and to evaluate its interaction with other prognostic factors.

## Figures and Tables

**Table 1:** Baseline characteristics.

		All	OS < 2 Years	%	OS ≥ 2 Years	%	*p*-Value
Patients		49	32		17		
Sex							
	Male	42	28	87.5	14	82.4	0.681
	Female	7	4	12.5	3	17.6	
Age (years)							
	<75	38	23	71.9	15	88.2	0.287
	≥75	11	9	28.1	2	11.8	
Median (range)		66 (29–84)	67.5 (29–84)		64 (40–80)		0.366
BMI							
	<18.5	24	17	53.1	7	41.2	0.551
	≥18.5	25	15	46.9	10	58.8	
	Median (range)	18.5 (12.6–29.8)	18.3 (12.6–22.6)		19.0 (14.7–29.8)		0.298
ECOG							
	PS 0–1	36	20	62.5	16	94.1	0.020
	PS 2–3	13	12	37.5	1	5.9	
Primary tumor site							
	Nasopharynx						0.312
	Oropharynx	12	8	25.0	4	23.5	
	Hypopharynx	13	10	31.2	3	17.6	
	Larynx	2	1	3.1	1	5.9	
	Oral cavity	10	6	18.8	4	23.5	
	Sinonasal tract	8	3	9.4	5	29.4	
	Others	4	4	12.5	0	0.0	
Treatment target site							
	Locoregional recurrence	35	22	68.8	13	76.5	0.743
	Distant metastasis	14	10	31.2	4	23.5	
Presence of diabetes mellitus							
	Yes	12	8	25.0	4	23.5	1.000
	No	37	24	75.0	13	76.5	
History of radiotherapy							
	Yes	36	23	71.9	13	76.5	1.000
	No	13	9	28.1	4	23.5	
Platinum-refractory carcinoma							
	Yes	39	27	84.4	14	82.4	1.000
	No	9	5	15.6	3	17.6	
Previous exposure to cetuximab							
	Yes	20	14	43.8	6	35.3	0.761
	No	29	18	56.2	11	64.7	
Albumin							
	<3.5 mg/dL	18	15	46.9	2	11.8	0.006
	≥3.5 mg/dL	31	17	53.1	15	88.2	
	Median (range)	3.6 (1.6–4.8)	3.4 (1.6–4.8)		3.8 (2.7–4.5)		0.020
NLR							
	<5	27	16	50.0	11	64.7	0.378
	≥5	22	16	50.0	6	35.3	
	Median (range)	4.4 (1.1–46)	4.9 (1.1–46)		3.8 (1.3–7.8)		0.130
NER							
	<32	24	12	37.5	12	70.6	0.007
	≥32	25	20	62.5	5	29.4	
	Median (range)	35 (5.5–850)	41.7 (5.5–850)		22.0 (7.5–320)		0.030

Abbreviations: BMI, body mass index; ECOG PS, Eastern Cooperative Oncology Group Performance Status; NLR, neutrophil-to-lymphocyte ratio; NER, neutrophil-to-eosinophil ratio; OS, overall survival.

**Table 2:** Treatment response in the overall population and subgroups.

	All		OS < 2 years		OS ≥ 2 Years	
Patients	49		32		17	
BOR (%)						
CR	7	(14.3)	0	(0.0)	7	41.2
PR	10	(20.4)	2	(6.3)	8	47.1
SD	5	(10.2)	3	(9.4)	2	11.8
PD	27	(55.1)	27	(84.4)	0	0.0
ORR (CR + PR)	17	(58.6)	2	(6.3)	15	(88.2)
DCR (CR + PR + SD)	22	(44.9)	5	(15.6)	17	(100.0)

Abbreviations: BOR best overall response; CR, complete response; DCR disease control rate; ORR objective response rate; OS, overall survival; PD, progressive disease; PR, partial response; SD, stable disease.

**Table 3:** Incidence of immune-related adverse events in the overall population and subgroups.

Category	All Cases		OS < 2 Years		OS ≥ 2 Years	
Number	49		32		17	
Case of irAE (+)	Any	Grade 3–5	Any	Grade 3–5	Any	Grade 3–5
Hypothyroidism	8	0	3	0	5	0
Hypoparathyroidism	4	0	2	0	2	0
Enterocolitis	0	1	0	0	0	1
Dermatitis	1	0	0	0	1	0
Number of events	13	1	5	0	8	1
Number of patients with any event	12		5		7	
Occurrence rate (%)	28.9		15.6		41.2	
			*p* = 0.0769			

irAE, immune-related adverse event.

**Table 4:** Univariate and multivariate logistic regression analysis to identify factors associated with long survival (OS ≥ 2 years) in all patients.

	Univariate			Multivariate		
	OR	95% CI	*p*-Value	OR	95% CI	*p*-Value
Age						
≥75 (Ref. <75)	0.341	0.0645–1.8	0.205			
Sex						
Male (Ref. Female)	0.667	0.176–2.60	0.626			
BMI						
≥18.5 (Ref. <18.5)	1.62	0.493–5.32	0.427			
ECOG-PS						
0–1 (Ref. 2–3)	12.4	1.47–106.0	0.021	11.9	1.21–116.0	0.034
Treatment target site						
Distant metastasis (Ref. loco lesional recurrence)	0.677	0.176–2.60	0.570			
Diabetes Mellitus						
Yes (Ref. No)	0.923	0.233–3.66	0.909			
Previous radiotherapy						
Yes (Ref. No)	0.786	0.202–3.06	0.729			
Platinum refractoriness						
Yes (Ref. No)	0.857	0.258–2.85	0.802			
Previous exposure to cetuximab						
Yes (Ref. No)	0.701	0.208–2.36	0.567			
Albumin						
≥3.5 (Ref. <3.5)	8.5	1.66–43.4	0.010	6.09	1.02–36.40	0.048
NLR						
≥5 (Ref. <5)	0.545	0.162–1.83	0.327			
NER						
<32 (Ref. ≥32)	6.2	1.63–23.60	0.007	4.27	0.934–19.50	0.061

Abbreviations: BMI, body mass index; ECOG-PS, Eastern Cooperative Oncology Group Performance Status; NLR, neutrophil-to-lymphocyte ratio; NER, neutrophil-to-eosinophil ratio.

## Data Availability

The datasets used and analyzed during the current study are available from the corresponding author upon reasonable request.
